# Effects of maternal methyl donor intake during pregnancy on ileum methylation and function in an intrauterine growth restriction pig model

**DOI:** 10.1186/s40104-023-00970-w

**Published:** 2024-02-04

**Authors:** Yan Lin, Jiangnan Wu, Yong Zhuo, Bin Feng, Zhengfeng Fang, Shengyu Xu, Jian Li, Hua Zhao, De Wu, Lun Hua, Lianqiang Che

**Affiliations:** Key Laboratory of Animal Disease-Resistance Nutrition, Animal Nutrition Institute, Sichuan Agricultural University, Chengdu, 611130 Sichuan China

**Keywords:** Ileum, Intrauterine growth restriction, Methyl donor, Methylation, Sows

## Abstract

**Background:**

Intrauterine growth retardation (IUGR) affects intestinal growth, morphology, and function, which leads to poor growth performance and high mortality. The present study explored whether maternal dietary methyl donor (MET) supplementation alleviates IUGR and enhances offspring’s growth performance by improving intestinal growth, function, and DNA methylation of the ileum in a porcine IUGR model.

**Methods:**

Forty multiparous sows were allocated to the control or MET diet groups from mating until delivery. After farrowing, 8 pairs of IUGR and normal birth weight piglets from 8 litters were selected for sampling before suckling colostrum.

**Results:**

The results showed that maternal MET supplementation tended to decrease the IUGR incidence and increased the average weaning weight of piglets. Moreover, maternal MET supplementation significantly reduced the plasma concentrations of isoleucine, cysteine, urea, and total amino acids in sows and newborn piglets. It also increased lactase and sucrase activity in the jejunum of newborn piglets. MET addition resulted in lower ileal methionine synthase activity and increased betaine homocysteine *S*-methyltransferase activity in the ileum of newborn piglets. DNA methylation analysis of the ileum showed that MET supplementation increased the methylation level of DNA CpG sites in the ileum of newborn piglets. Down-regulated differentially methylated genes were enriched in folic acid binding, insulin receptor signaling pathway, and endothelial cell proliferation. In contrast, up-regulated methylated genes were enriched in growth hormone receptor signaling pathway and nitric oxide biosynthetic process.

**Conclusions:**

Maternal MET supplementation can reduce the incidence of IUGR and increase the weaning litter weight of piglets, which may be associated with better intestinal function and methylation status.

## Background

Intrauterine growth restriction (IUGR) with a lower birth weight has been associated with a higher incidence of perinatal morbidity and mortality in humans and domestic animals. IUGR has been shown to affect 5%–8% of human neonates [1] and 10%–20% of neonates in the pig industry [2]. The occurrence of IUGR is likely due to the impaired utero-placental function or insufficient nutrition, in which nutrients in the fetus are directed to the vital tissues, such as the brain and heart, while limiting nutrient distribution to other tissues, such as the lungs [3], intestine [4, 5], and skeletal muscle [6]. Furthermore, IUGR-afflicted neonates have a lower small intestinal weight and mucosa weight [7], as well as impaired intestinal morphology, digestion and absorption of nutrients, and barrier function [8–10], and lower diversity of intestinal microbiota [11]. Our recent study showed that IUGR leads to disparate DNA methylation in the intestine [12], which may play a crucial role in the impairment of IUGR intestine.

Previous studies have shown that maternal nutrition can lead to permanent phenotypic changes in the offspring via epigenetic modifications, such as DNA methylation and histone acetylation. Maternal high-fat diet induced sex-specific epigenetic and metabolic changes in the rat offspring [13], while maternal methyl donor (MET) supplementation increased β-cell numerical density and improved insulin sensitivity in twin lambs affected by IUGR [14]. Our previous study also found that dietary MET supplementation during gestation enhanced the birthweight and postnatal growth rate of the offspring, which was associated with an increased expression of the *IGF-1* gene and altered DNA methylation of the *IGF-1* gene promoter in the liver [15]. Furthermore, research conducted on humans has revealed that women with higher methyl group intake showed higher DNA methylation in the third trimester and not in the earlier phases of pregnancy [16]. Similarly, restricting the supply of specific B vitamins (i.e., B_12_ and folate) and methionine as part of the periconceptional diet in mature female sheep led to adult offspring that were heavier and displayed widespread epigenetic alterations in DNA methylation [17]. In addition, maternal choline supplementation as a MET source modulated placental inflammation and angiogenesis in mice [18]. In contrast, MET deficiency during pregnancy impaired fetal gastric ghrelin cell organization and decreased plasma ghrelin levels in rats [19]. Research has shown that dietary supplementation with MET altered the methylation of fibroblast growth factor 21 [20] and insulin-like growth factor-1 in fetal liver in an IUGR rat model [21]. A significant differential methylation was observed in the promoter region of H19 in the liver among the IUGR group participants, whereas the MET diet was associated with hypermethylation in the IUGR group rats [22]. However, to the best of our knowledge, the effects of maternal MET supplementation on gastrointestinal development and the underlying mechanism remain unclear in porcine models of IUGR.

Therefore, we hypothesized that maternal MET supplementation affects the intestinal morphology and function via epigenetic modification. Using a sow receiving MET supplements throughout gestation, global and site-specific DNA methylation of the newborn intestine was established and the parameters related to intestinal function and one-carbon metabolism were determined.

## Materials and methods

### Animals and experimental design

All animal experimental procedures were approved by the Animal Care and Use Committee of the Animal Nutrition Institute, Sichuan Agricultural University and were in accordance with the current animal protection laws (Ethics Approval Code: SCAUAC202216–7). A total of 40 Landrace × Yorkshire sows (parity 3–6) with backfat thickness of 18 ± 0.32 mm were artificially inseminated and randomly allocated into two groups: one receiving the control diet (CON) and one receiving the MET-supplemented diet throughout gestation. The MET supplement consisted of 3 g/kg betaine (96% betaine hydrochloride, Skystone Feed Co., Ltd., Jiangsu, China), 400 mg/kg choline (50% choline chloride; Enbei Group, Shangdong, China), 15 mg/kg folic acid (80%, Shengda Bio-Pharm Co., Ltd., Zhejiang, China), and 150 μg/kg vitamin B_12_ (CSPC Pharmaceutical Group Co., Ltd., Hebei, China). As shown in Table 1, the CON diet for gestating and lactating sows was formulated based on the nutrient requirements for swine provided by the National Research Council (2012) [23].

**Table 1:** Dietary formulation and nutrient levels for pregnant sows

Ingredients, %	CON	MET
Corn	19.85	19.85
Barley	35.00	35.00
Wheat bran	25.00	25.00
Sorghum	5.00	5.00
Sunflower seed meal	10.05	10.05
L-Lysine·HCl (98%)	0.34	0.34
DL-Methionine (98.5%)	0.04	0.04
L-Threonine (98%)	0.06	0.06
Choline chloride (50%)	0.29	0.29
Limestone	1.84	1.84
Monocalcium phosphate	0.51	0.51
NaCl	0.50	0.50
Vitamin and mineral premix^a^	0.32	0.32
Carriers^b^	1.20	0.70
Methyl donors		0.50
Total	100.00	100.00
Nutrient levels^c^		
Net energy, Mcal/kg	2.05	2.05
Crude protein, %	13.00	13.00
Crude fiber, %	6.40	6.40
Calcium, %	0.87	0.87
Total phosphorus, %	0.51	0.51
Total lysine, %	0.71	0.71
Total methionine, %	0.25	0.25
Total threonine, %	0.59	0.59
Total tryptophan, %	0.17	0.17
Folic acid, mg/kg	1.23	16.23
Vitamin B_12_, μg/kg	14.20	164.20
Choline, mg/kg	1,073	1,473
Betaine, mg/kg	0.00	3,000

^a^Vitamin mixture supplied the following amounts of vitamins/kg of complete diet: 3,786 IU vitamin A; 757 IU vitamin D_3_; 42 IU vitamin E; 1.0 mg vitamin B_1_; 3.5 mg vitamin B_2_; 1.0 mg vitamin B_6_; 15.0 μg vitamin B_12_; 10 mg niacin; 1.2 mg folate; 1,073 mg choline. Mineral mixture per kilogram of diet contained 76 mg Fe; 9.5 mg Cu; 95 mg Zn; 24 mg Mn; 0.15 mg I; 0.15 mg Se

^b^The carriers were complex of rice bran and corn starch

^c^Calculated according to China Feed Database (2014) [24]

### Feeding management

All sows were fed 2.1 kg/d of feed starting on d 1 until d 90 of gestation and 2.8 kg of feed/d starting on d 91 until farrowing, followed by providing free access to the lactation diet thereafter. The ambient temperature for sows was maintained at 19–21 °C and the humidity was 60%–70%. The sows were transferred to the lactating sow house 1 week before delivery. The temperature of the delivery room was controlled at approximately 25 °C. After farrowing, litters were adjusted to be of similar size by cross-fostering within treatment. Sows were fed the same lactation diet three times per day (08:00, 16:00, and 20:00 h) for a 25-d period. Standard husbandry practices were used during the experimental period, which included iron supplementation (injection of 200 mg of Fe as dextran iron), castration (on d 5 postpartum), and vaccinations. Sows had free access to drinking water and the pens were cleaned daily and disinfected twice a week.

### Sample collection and analysis

On the farrowing day, 10 mL of blood was collected from the ear vein of sows using heparin as an anticoagulant and then centrifuged at 3,500 r/min at 4 °C for 15 min. The supernatant plasma was stored at −20 °C. After farrowing, 8 pairs of IUGR and normal birth weight (NBW) piglets from 8 litters were selected for slaughtering and sampling before suckling colostrum. Piglets with birth weight near the mean litter birth weight (SD: 0.5) were deemed as NBW, whereas those with lower birth weight (at least 1.5 SD) were considered as IUGR according to our previous study [25]. The body weight (BW) of the NBW and IUGR piglets was 1.32 ± 0.15 kg and 0.86 ± 0.12 kg, respectively. Intestinal tissue was then removed, the jejunum and ileum were separated, and approximately 2 cm of the middle section of each intestine was cut and fixed in a 4% paraformaldehyde solution for intestinal morphology analysis. In addition, the middle segments of the jejunum and ileum were collected, snap-frozen in liquid nitrogen, and stored at −80 °C for parameter measurements.

### Reproductive performance

The total number of piglets born (alive, stillborn, and mummy) was recorded and the individual BW of piglets born alive was noted after farrowing and before suckling colostrum. The IUGR rate was also calculated. The number of weaned piglets and their litter weight were documented. Additionally, the weaning survival rate, daily weight gain, and litter weight gain were calculated. The daily feed intake of the sows was recorded every week, and the average feed intake during the lactation period was calculated.

### Jejunum histology

Morphological samples of the jejunum were processed for paraffin preparation for hematoxylin and eosin staining as described in our previous study [25]. A light microscope (Olympus Bx51, Olympus, Tokyo, Japan) connected to Image Pro Plus 6.0 (Media Cybernetics, Inc., Rockville, MD, USA) software was used to measure 10 villous height (VH) as well as the corresponding crypt depth (CD). The V/C ratio was equal to the VH value divided by the CD value. Goblet cells within 10 randomly selected villi were counted at 400× magnification.

### Amino acid profile assay in plasma

Amino acid samples from the plasma of sows and piglets were mixed with 10% sulfosalicylic acid at a ratio of 1:2, vortexed, incubated at 4 °C for 30 min, and centrifuged at 10,000 r/min for 10 min. The supernatant was filtered into a bottle and then evaluated using an amino acid analyzer (L-8900, Hitachi High Tech, Tokyo, Japan).

### Digestive enzyme activities

The activities of disaccharidases, including maltase, sucrose, and lactase, were measured according to the method previously described by Che et al. [26]. Briefly, jejunal samples (0.2–0.5 g) were weighed, homogenized, and centrifuged at 3,000 × *g* for 10 min. The supernatant was then collected for the enzyme assay. Total protein samples were extracted and their concentration was determined based on the bicinchoninic acid assay procedure (Solarbio, Inc., Beijing, China). Disaccharidase activity was measured using commercial kits according to the manufacturer’s instructions (Nanjing Jiancheng Bioengineering Institute, Nanjing, China) and expressed as U/mg protein.

### Enzyme activity in one-carbon metabolism

The activities of methionine synthase, DNA methyltransferase (DNMT), and betaine homocysteine *S*-methyltransferase (BHMT) were measured using commercial ELISA kits according to the manufacturer’s instructions (Jiangsu Enzyme Immune Industrial Co., Ltd., Jiangsu, China). Briefly, jejunal samples (0.2–0.5 g) were weighed, homogenized at 4 °C, and centrifuged at 3,000 × *g* for 10 min. The supernatants were collected and analyzed using 3,3′,5,5′-tetramethylbenzidine as the chromogen substrate. The absorbance value was measured with an enzyme marker (SpectraMax 190, Molecular Devices, CA, USA) at a wavelength of 450 nm to calculate sample activity.

### Global DNA methylation analysis and reduced representation bisulfite sequencing (RRBS) library construction and sequencing

Sixteen ileum samples from the IUGR/NBW pairs (1–8 from each group) were selected for RRBS analysis. About 1 μg of genomic DNA mixed with unmethylated lambda DNA was digested by MspI enzyme for 16 h at 37 °C. After digestion, the libraries were constructed using the Illumina Pair-End protocol with some modifications. Briefly, purified digested DNA was subsequently treated with a mix of T4 DNA polymerase, Klenow Fragment, and T4 polynucleotide kinase to repair, blunt, and phosphorylate ends. Next, blunt DNA was 3′-adenylated using Klenow Fragment (3′-5′ exo-), followed by ligation to adaptors synthesized with 5′-methylcytosine instead of cytosine using T4 DNA ligase. After each step, DNA was purified using the MinElute PCR Purification Kit (Qiagen, Hilden, Germany). The EZ DNA Methylation-Gold Kit (Cat. No. D5006, Zymo Research, Irvine, CA, USA) was used to convert unmethylated cytosine into uracil according to the manufacturer’s instructions. Finally, PCR was carried out in a final reaction volume of 50 μL consisting of 20 μL of adapter ligated DNA, 4 μL of 2.5 mmol/L dNTPs, 5 μL of 10× buffer, 0.5 μL of JumpStart™ Taq DNA Polymerase, 2 μL of PCR primers, and 18.5 μL of water. The thermal cycling program was as follows: denaturation at 94 °C (1 min), 12 cycles of annealing at 94 °C (10 s), 62 °C (30 s), 72 °C (30 s), and elongation at 72 °C (5 min). PCR products were maintained at 12 °C. Before analysis with the Illumina sequencing platform, the size-selected library was analyzed using the Bioanalyzer analysis system (Agilent, Santa Clara, CA, USA) and quantified using real-time PCR.

### Raw data filtration and data processing

Raw sequencing data were processed using the Illumina basecalling pipeline. Adapter sequences were trimmed using Cutadapt. The parameter settings were “-a AGATCGGAAGAGC -m 35 -n 2.” The cleaned reads were mapped back to the genome using BSMAP software version 2.90 [27]. The parameter settings were “-n 0 -v 0.08 -g 1”. Methylation ratios were extracted from the BSMAP output (SAM) using a Python script (methratio.py), which was distributed with the BSMAP package. Only unique mapped reads were used to calculate the methylation ratios. Only cytosines in a CpG context with sufficient sequencing depth (≥ 5× coverage) were retained for further analysis. Differentially methylated regions (DMRs) were detected using metilene [28] in de novo mode among the CpG sites with at least 5× coverage. The parameter settings were “--mincpgs 5 --minMethDiff 0.1 --mtc 1 -X 1 -Y 1 -v 0.7”. The detected DMRs were filtered according to the following standards: (1) *Q*-value < 0.05; (2) methylation level difference > 0.1; (3) CpG number contained in the DMR > 5; and (4) DMR length > 50 bp.

### Statistical analysis

Split-plot experimental design was used in the present study. There were two experimental factors: maternal diet effect was the main factor and birth weight effect in piglets was the secondary factor. The significance of the data was analyzed using SAS 9.4 software. The normality test was carried out before the statistical data analysis. The data on reproductive performance, colostrum composition, and plasma amino acid concentration of sows were analyzed using the independent samples *t*-test. The incidence of IUGR was determined using the Chi-squared (χ^2^) test. Other statistical parameters were evaluated using a mixed model procedure. When the main effect was significant, multiple comparisons were carried out using the LSD method. The results were expressed as the average value and combined standard error. The difference was significant when *P* < 0.05, while 0.05 ≤ *P* ≤ 0.10 was considered to indicate a tendency.

## Results

### Reproductive performance

Dietary supplementation with MET in gestating sows did not markedly affect the litter size and number born alive, or birth weight. However, dietary supplementation with MET tended to decrease the IUGR incidence (− 4.50%, *P* = 0.08, Table 2) and increased the feed intake of sows during lactation (+ 4.31%, *P* = 0.01). Accordingly, the litter weight (+ 18.59%, *P* = 0.01) and BW of piglets on d 25 of lactation (+ 9.86%, *P* = 0.05) were increased.

**Table 2:** Effect of maternal MET supplementation on reproductive performance of sows

Item	CON	MET	SEM	*P*-value
Farrowing duration, min	346.50	320.70	21.29	0.56
Farrowing interval, min	21.61	16.71	1.33	0.07
ADFI during lactation, kg/d	6.96	7.26	0.06	0.01
Total born, n	18.70	18.74	0.53	0.97
Born alive, n	17.70	17.80	0.47	0.84
NBW, n	15.65	16.63	0.45	0.29
IUGR, n	2.05	1.26	0.26	0.13
Stillborn, n	0.85	0.63	0.14	0.45
Born mummy, n	0.15	0.21	0.06	0.63
Weaned piglets, n	12.60	13.26	0.26	0.128
IUGR incidence, %	10.76	6.26	1.67	0.08
Average birth weight, kg	1.16	1.23	0.03	0.21
Average litter weight at birth, kg	21.55	22.74	0.57	0.31
Average weaning weight, kg	6.39	7.02	0.16	0.05
Average weaning litter weight, kg	72.40	85.86	2.63	0.01
ADG during lactation, g/d	250.00	280.00	10	0.11

*CON* Control group, *MET* Methyl donors’ group, *ADFI* Average daily feed intake, *ADG* Average daily gain

*n* = 20 in CON group and *n* = 20 in MET group

### Plasma amino acid concentration

Dietary supplementation with MET in gestating sows decreased the plasma levels of Val (*P* < 0.01), Ile (*P* = 0.01), Leu (*P* = 0.03), Phe (*P* = 0.01), Cys (*P* < 0.01), urea (*P* = 0.01), and total amino acids (*P* = 0.01) on farrowing day (Table 3). In addition, plasma concentrations of Thr (*P* = 0.01), Ser (*P* = 0.02), Lys (*P* = 0.09), Glu (*P* = 0.07), Tyr (*P* = 0.07), Asp (*P* = 0.05), and urea (*P* = 0.07) in the IUGR piglets were higher than those in the NBW piglets (Table 4). On the other hand, Cys level was lower (*P* = 0.02) than that in the NBW piglets. Compared to the CON group, MET supplementation during pregnancy increased the level of plasma His level (*P* = 0.01) and decreased the levels of plasma Ile (*P* = 0.02), Ala (*P* < 0.01), Ser (*P* = 0.04), Cys (*P* < 0.01), urea (*P* < 0.01), and total amino acid (*P* < 0.01) levels in newborn piglets.

**Table 3:** Effect of maternal MET supplementation on plasma amino acid composition of sows

Item	CON	MET	SEM	*P*-value
Essential amino acids, nmol/mL				
Lys	291.6	203.9	28.70	0.13
Met	62.54	60.89	4.12	0.85
Thr	139.10	79.50	15.66	0.054
Val	292.10	194.20	18.68	0.005
Ile	133.10	99.57	7.12	0.01
Leu	252.60	176.60	17.91	0.03
Phe	204.00	145.00	12.10	0.01
Trp	23.98	28.65	2.23	0.31
His	83.40	71.55	3.66	0.11
Arg	294.00	222.00	27.49	0.20
Non-essential amino acids, nmol/mL				
Glu	85.91	99.30	6.23	0.29
Gly	710.10	817.30	44.50	0.24
Ala	447.20	524.10	27.28	0.16
Cit	119.60	139.40	9.05	0.29
Ser	13.80	8.87	2.18	0.27
Cys	37.49	13.79	4.55	0.005
Tyr	134.60	130.30	8.52	0.81
Orn	96.06	77.94	7.36	0.23
Pro	407.00	415.40	29.45	0.90
Tau	122.60	129.70	5.62	0.54
Asp	5.79	4.46	0.62	0.30
Urea	4,472	3,238	254.94	0.01
Total	9,047	7,169	396.19	0.01

*CON* Control group, *MET* Methyl donors’ group

^a–c^*P* < 0.05 between different superscripts within the same line, *n* = 10 in each group

**Table 4:** Effect of maternal MET supplementation on plasma amino acid composition of neonatal piglets

Item	Treatments				SEM	*P*-value		
	CON_IUGR	CON_NBW	MET_IUGR	MET_NBW		BW	MET	BW × MET
Essential amino acids, nmol/mL								
Lys	390.21^a^	205.11^b^	305.91^ab^	363.69^a^	27.55	0.02	0.18	< 0.01
Met	36.38	26.00	20.96	26.95	6.27	0.66	0.23	0.23
Thr	316.97^a^	155.33^c^	185.78^bc^	226.73^b^	20.11	0.02	0.24	< 0.01
Val	265.95	282.08	246.00	273.10	12.45	0.11	0.28	0.68
Ile	54.28^a^	36.21^b^	28.63^b^	31.09^b^	5.94	0.22	0.02	0.11
Leu	122.00	66.90	108.75	115.05	10.35	0.22	0.37	0.12
Phe	98.93^a^	69.75^b^	72.09^b^	84.30^ab^	9.82	0.38	0.52	0.04
Trp	1.24	0.73	0.98	0.63	0.31	0.28	0.22	0.56
His	58.50^b^	20.63^c^	54.32^b^	81.13^a^	4.63	0.48	< 0.01	< 0.01
Arg	18.73	42.95	19.88	26.61	9.75	0.13	0.45	0.39
Non essential amino acids, nmol/mL								
Glu	264.83	150.13	190.99	225.98	24.57	0.96	0.82	0.09
Gly	1,662.69^a^	1,289.57^bc^	1,192.03^c^	1,544.49^ab^	160.35	0.93	0.34	< 0.01
Ala	1,062.73^a^	786.88^bc^	682.09^c^	860.06^b^	28.16	0.38	< 0.01	< 0.01
Cit	138.83	123.04	103.31	128.10	21.07	0.83	0.48	0.34
Ser	317.18^a^	215.60^b^	223.35^b^	238.63^b^	25.34	0.02	0.04	< 0.01
Cys	12.18^bc^	42.45^a^	15.71^b^	4.56^c^	2.96	0.02	< 0.01	< 0.01
Tyr	92.72	47.08	68.27	61.35	15.50	0.07	0.71	0.17
Orn	155.89^a^	78.90^b^	98.64^b^	168.45^a^	13.06	0.80	0.27	< 0.01
Pro	276.75^a^	185.08^b^	185.21^b^	265.75^a^	20.67	0.83	0.84	< 0.01
Tau	288.55^a^	178.99^b^	167.63^b^	236.45^ab^	31.64	0.52	0.32	< 0.01
Asp	53.33^ab^	32.35^b^	56.55^a^	46.97^ab^	1.52	0.05	0.24	0.45
Urea	5,103^a^	3,673^b^	2,864^c^	3,394^bc^	310.46	0.07	< 0.01	< 0.01
Total	12,175^a^	9,792^bc^	8,535^c^	10,108^b^	341.19	0.48	< 0.01	< 0.01

*CON_IUGR* CON group with IUGR piglets, *CON_NBW* CON group with NBW piglets, *MET_IUGR* MET group with IUGR piglets, *MET_NBW* MET group with NBW piglets

^a–c^*P* < 0.05 between different superscripts within the same line, *n* = 10 in each group

### Jejunal disaccharidase activity

MET supplementation significantly increased the lactase and sucrose activity (*P* < 0.01, Table 5) and decreased the total protein level (*P* < 0.01) in the jejunum of newborn piglets compared to those in the CON group.

**Table 5:** Effect of maternal MET supplementation on the activities of jejunal disaccharidase in newborn piglets

Item	Treatments				SEM	*P*-value		
	CON_IUGR	CON_NBW	MET_IUGR	MET_NBW		BW	MET	BW × MET
Total protein, g/L	4.68^a^	4.57^a^	2.97^b^	3.08^b^	0.33	0.99	< 0.01	0.73
Lactase, U/mg protein	38.24^c^	56.73^bc^	86.63^a^	66.50^ab^	3.34	0.92	< 0.01	0.02
Sucrase, U/mg protein	54.78^b^	52.13^b^	85.44^a^	75.71^a^	1.97	0.15	< 0.01	0.40
Maltase, U/mg protein	15.33	12.65	14.37	12.19	2.66	0.39	0.80	0.93

*CON_IUGR* CON group with IUGR piglets, *CON_NBW* CON group with NBW piglets, *MET_IUGR* MET group with IUGR piglets, *MET_NBW* MET group with NBW piglets

^a–c^*P* < 0.05 between different superscripts within the same line, *n* = 10 in each group

### Ileum morphology

Piglets in the CON_IUGR group had numerically lower VH (by 24%) and CD (by 19%) values compared to those in the CON_NBW group (Table 6). Maternal MET intake had no significant effect on VH, CD, and goblet cell density of the ileal tissue (*P* > 0.05), whereas birth weight and MET supplementation had an interactive effect on CD (*P* < 0.1) compared to the CON group.

**Table 6:** Effect of maternal MET supplementation on the ileum morphology of newborn piglets

Item	Treatments				SEM	*P*-value		
	CON_IUGR	CON_NBW	MET_IUGR	MET_NBW		BW	MET	BW × MET
Villus height, μm	531.74	657.64	561.61	572.48	43.03	0.16	0.56	0.24
Crypt depth, μm	59.99	71.43	74.40	68.65	4.40	0.54	0.21	0.07
Villus height/Crypt depth	8.79	9.57	8.24	9.95	0.78	0.13	0.92	0.57
Goblet cell density, number/mm^2^	574.25	541.35	548.43	563.84	35.75	0.83	0.97	0.54

*CON_IUGR* CON group with IUGR piglets, *CON_NBW* CON group with NBW piglets, *MET_IUGR* MET group with IUGR piglets, *MET_NBW* MET group with NBW piglets

*n* = 10 in each group

### One-carbon metabolism-related enzyme activity in the ileum

Interestingly, methionine synthase activity in the ileum of NBW piglets was lower than that in the IUGR piglets (− 18.52%, *P* = 0.09, Table 7). MET supplementation in the gestation period significantly decreased the activity of methionine synthase in the ileum of newborn piglets (− 25.41%, *P* = 0.03) and increased the activity of BHMT (+ 10.40%, *P* = 0.02) compared to the CON group.

**Table 7:** Effect of maternal MET supplementation on the activity of one -carbon metabolism enzyme in the ileum of newborn piglets

Item	Treatments				SEM	*P*-value		
	CON_IUGR	CON_NBW	MET_IUGR	MET_NBW		BW	MET	BW × MET
MetS, U/L	222.94^a^	199.21^ab^	188.59^ab^	148.02^b^	22.15	0.09	0.03	0.65
DNMTs, U/L	23.71	24.82	25.69	27.22	1.34	0.37	0.14	0.88
BHMT, U/L	265.91^b^	263.75^b^	299.18^a^	285.54^ab^	13.08	0.47	0.02	0.60

*CON_IUGR* CON group with IUGR piglets, *CON_NBW* CON group with NBW piglets, *MET_IUGR* MET group with IUGR piglets, *MET_NBW* MET group with NBW piglets, *MetS* Methionine synthase, *DNMTs* DNA methyltransferases, *BHMT* Betaine homocysteine methyltransferase

^a,b^*P* < 0.05 between different superscripts within the same line, *n* = 10 in each group

### Genome-wide CpG methylation in relation to genomic features

Approximately 449.1 million reads of 150-bp paired-end RRBS DNA methylome data were generated, corresponding to an average of 25.0 million sequence reads per RRBS sample. A significant increase in DNA methylation was found in the MET_NBW group compared to that in the CON_IUGR and CON_NBW groups. Although DNA methylation of MET/NBW increased by 2.4% compared to that of MET/IUGR, there was no significant methylation alteration in piglets of different birth weight (Table 8).

**Table 8:** Effect of maternal MET supplementation on the DNA methylation level of ileum in newborn piglet

Item	Treatments				SEM	*P*-value		
	CON_IUGR	CON_NBW	MET_IUGR	MET_NBW		BW	MET	BW × MET
5× Merged CG_MeanRate, %	65.00	64.47	65.32	65.23	0.94	0.62	0.39	0.73
5× CG_MeanRate, %	63.08^b^	62.95^b^	64.32^ab^	65.87^a^	1.11	0.03	0.43	0.36
Promoter, %	31.45	30.69	31.24	30.90	0.64	0.22	1.00	0.63

*CON_IUGR* CON group with IUGR piglets, *CON_NBW* CON group with NBW piglets, *MET_IUGR* MET group with IUGR piglets, *MET_NBW* MET group with NBW piglets

CG_MeanRate means the average methylation rate of CG type cytosine

^a,b^*P* < 0.05 between different superscripts within the same line, *n* = 4 in each group

**Fig. 1:**
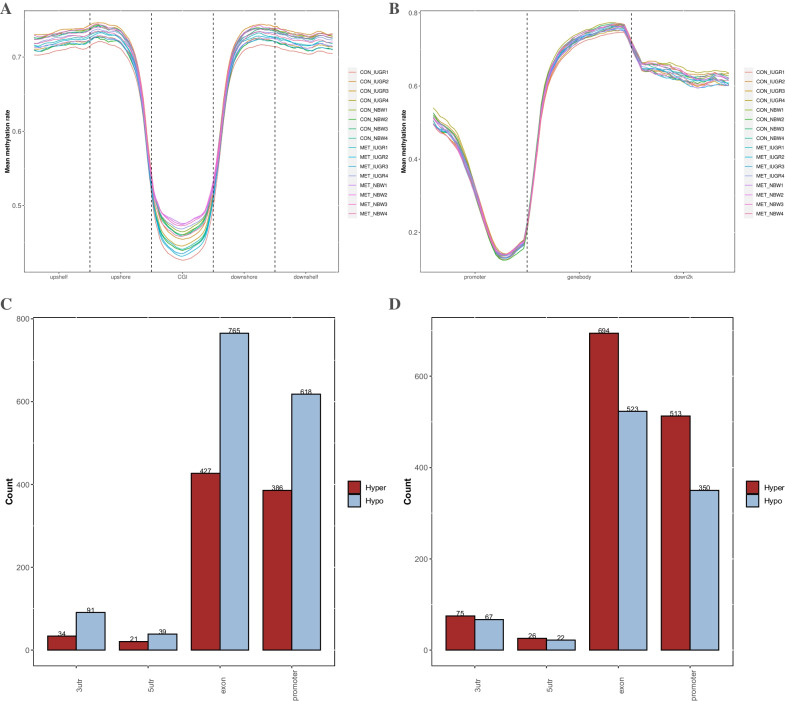
Genome-wide CpG sites methylation in relation to genomic features and IUGR and MET-induced DMCs. **A **and **B** Average CpG methylation levels for various genomic features. Data are expressed as mean ± SEM. **C** and **D** CpG methylation levels in relation to genic regions and element pattern

Profiling CpG methylation patterns (Fig. 1A) revealed that the piglets’ ileum in the CON_IUGR group was hypomethylated compared to that in the CON_NBW group. However, a significant increase in CpG methylation level was observed in the ileum after MET supplementation in the MET_IUGR and CON_IUGR groups (Fig. 1B). Consistently, lower CpG methylation levels were observed within CpG islands than within CpG island shores in all groups (Fig. 1C). Additionally, all samples displayed a similar dip in methylation centered at promoter sites (Fig. 1D), which gradually increased toward the gene bodies (Fig. 1D).

### Genomic distribution of cytosine methylation

Genomic methylation distribution was determined for the ileum. In the present study, genes that overlapped with the methylation peaks in the upstream 2-kb, downstream 2-kb, or gene body regions were termed methylated genes. Of these, 1,659 methylated genes were identified as differentially expressed genes in the CON_IUGR and CON_NBW groups (Table 9). They included 1,044 hypermethylated and 1,245 hypomethylated genes in the CON_IUGR group. A total of 1,887 methylated genes were identified as differentially expressed genes in the MET_IUGR and MET_NBW groups. These included 1,233 hypermethylated and 1,406 hypomethylated genes in the CON_IUGR group. Differential methylation sites were located in gene bodies and to a lesser extent in exons and promoters.

**Table 9:** Numbers of differentially methylated genes of different group in different gene regions

Element	CON_IUGR vs. CON_NBW-Hyper	CON_IUGR vs. CON_NBW-Hypo	MET_IUGR vs. MET_NBW-Hyper	MET_IUGR vs. MET_NBW-Hypo
3UTR	31	59	44	65
5UTR	18	36	38	44
CDS	278	385	311	457
CGI	502	544	510	558
Down2k	192	241	211	289
Downshelf	311	365	328	369
Downshore	305	340	328	344
Exon	318	461	381	546
Genebody	1,044	1,245	1,233	1,406
Promoter	261	427	333	507
Upshelf	320	359	331	371
Upshore	307	329	332	356

*CON_IUGR* CON group with IUGR piglets, *CON_NBW* CON group with NBW piglets, *MET_IUGR* MET group with IUGR piglets, *MET_NBW* MET group with NBW piglets

### BW and MET-induced DMR methylation profiling change

**Fig. 2:**
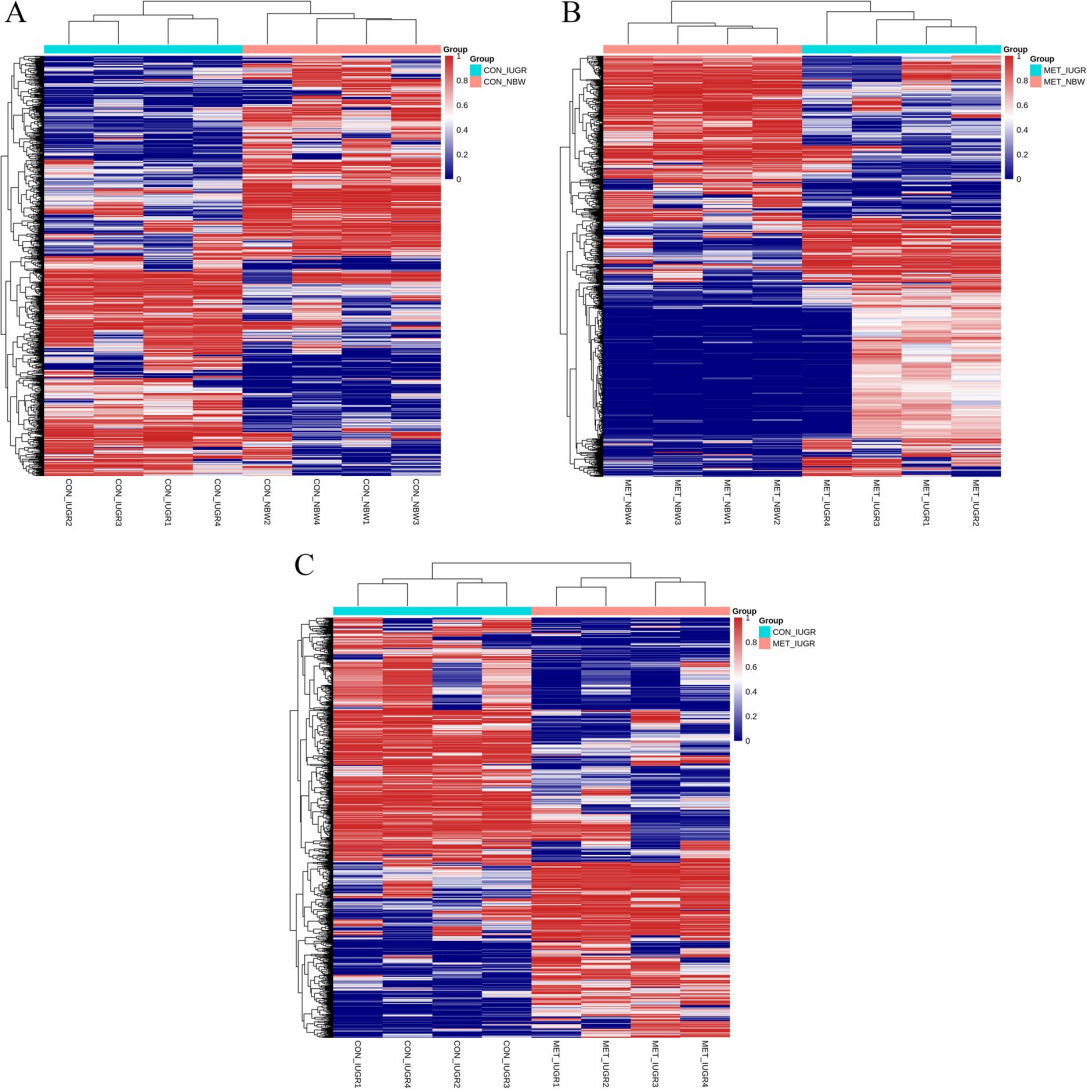
Heat map of the top 1,000 loci from piglet’s ileum. Each row in this heat map corresponds to data from a single locus, whereas each columns correspond to individual samples. **A** CON_IUGR and CON_NBW, **B** MET_IUGR and MET_NBW, **C** MET_IUGR and CON_IUGR. The branching dendrogram corresponds to the relationships among samples, as determined by clustering using these 1,000 sites. All figures show similar numbers of loci becoming relatively hyper- and hypomethylated (red to blue), respectively

Next, differentially methylated CpG (DMCs) were identified between the CON_IUGR vs. CON_NBW and the MET_IUGR vs. MET_NBW groups, respectively. The DMCs were based on a *Q* value of < 0.05 and methylation differences between the two groups of > 10%. A total of 80,265 DMCs were identified when evaluating the CON_IUGR and CON_NBW piglets’ ileum, including 33,000 hypermethylated and 47,265 hypomethylated DMCs. Next, 100,249 DMCs were identified when analyzing the MET_IUGR vs. MET_NBW piglets’ ileum, including 45,221 hypermethylated and 55,028 hypomethylated DMCs. Finally, 87,919 DMCs were found in a comparison of MET_IUGR vs. CON_IUGR piglets, including 47,883 hypermethylated and 40,036 hypomethylated DMCs. A heat map of the top 1,000 differentially methylated loci showed that this loci subset readily distinguished between BW and MET supplementation (Fig. 2).

### BW and MET-induced ileum DNA methylation changes

**Fig. 3:**
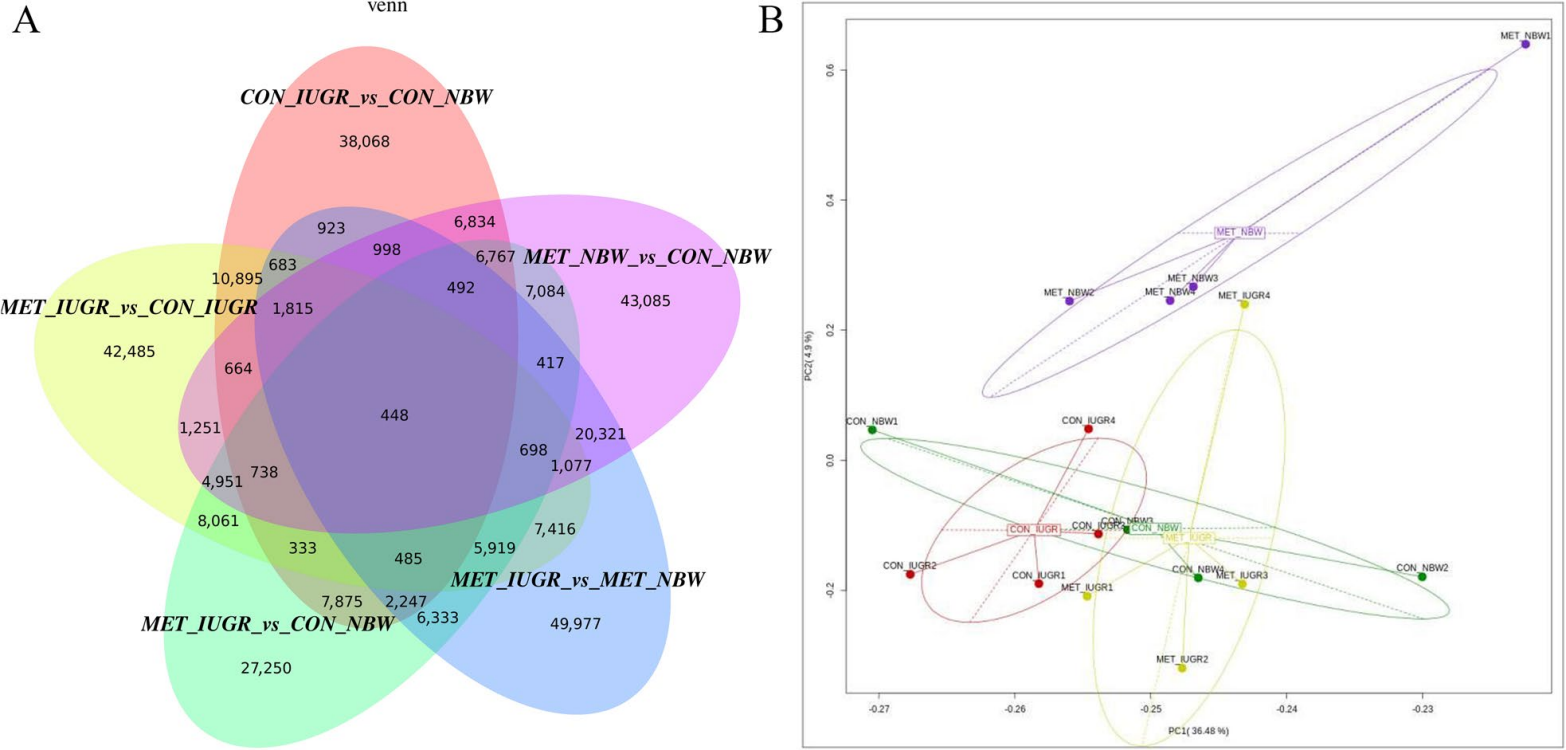
Summary of DMCs induced by body weight and MET supplementation. **A** Venn diagram showing the number of DMCs in the different groups piglets. It shows the result of the cross-matching genes with DMCs overlapping with respect to the different pairs. **B** Principal component analysis (PCA) showed differences in DNA methylation between groups

The study results showed that there was a significant difference in DNA methylation in the ileum of newborn piglets from the IUGR and NBW groups (Fig. 3A). Principal component analysis demonstrated a significant difference in DNA methylation in the ileum of IUGR piglets compared to the two groups of sows that were fed the control and methyl donor diets (Fig. 3B). There was a smaller difference in DNA methylation levels in the ileal tissue between the IUGR piglets from the methyl donor group and the NBW piglets from the CON group.

### BW and MET-induced differential methylation in chromosomes

**Fig. 4:**
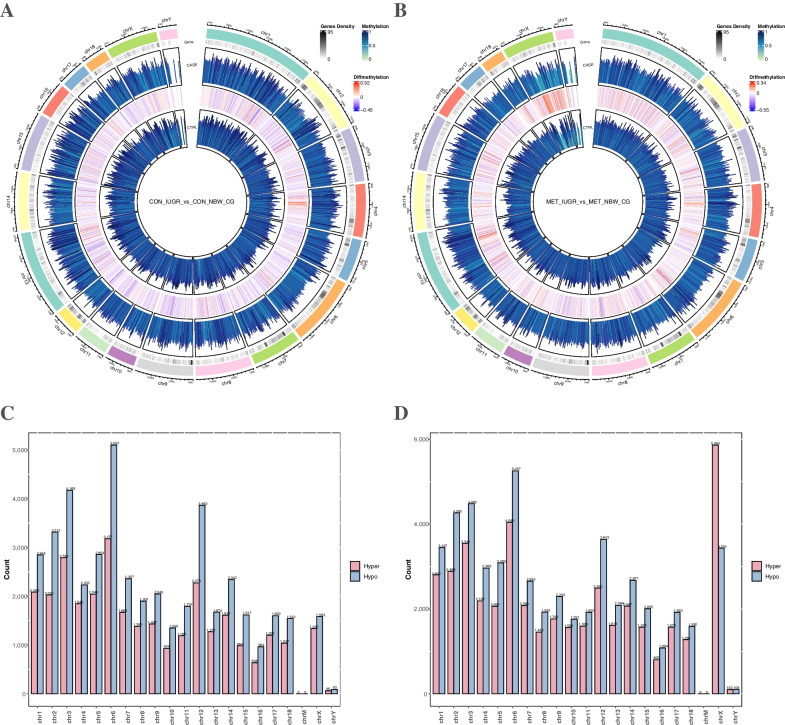
Circos plot of DMC methylation profiles of piglet’s ileum. **A** and **C** CON_IUGR vs. CON_NBW group; **B** and **D** MET_IUGR vs. MET_NBW ileum of piglets. Blue represents methylation level of DMCs in different group. Pink represents DMCs represent diffmethylation level of different group

Differential methylation in different groups was represented in a Circos plot covering all of the chromosomes. Circos plot represents differentially methylated regions between the groups (Fig. 4A and B). The outermost circle (shown in various colors) represents different chromosomes. Specific chromosome names are marked on the map. The second circle (black and white colored circles) represents gene density. Circle 3 (blue circle) represents the methylation level of the treatment group. Circle 4 represents the level of differential methylation, and the innermost circle represents the methylation level of the CON group. DMCs were located on chromosomes 1–3, 6, and 12 when comparing the CON_IUGR and CON_NBW groups (Fig. 4A and C). Chromosome 6 had the highest dimethylation level, including 3,177 hypermethylated and 5,097 hypomethylated DMCs. The DMCs were located on chromosomes X when comparing the MET_IUGR and MET_NBW groups. There were 5,864 hypermethylated and 3,434 hypomethylated DMCs (Fig. 4B and D), which showed that methylation level was increased in the ileum after MET supplementation.

### Gene ontology term analysis of DNA methylation

**Fig. 5:**
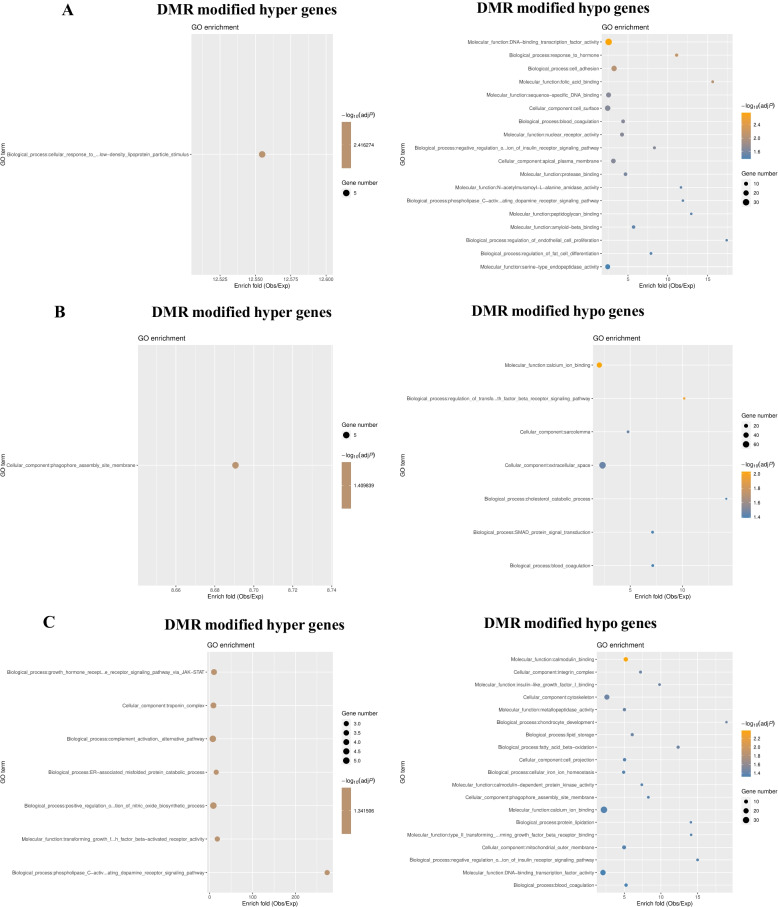
Representative enriched GO terms of DNA methylation driven genes in piglet’s ileum. **A** CON_IUGR vs. CON_NBW; **B** MET_IUGR vs. MET_NBW; **C** MET IUGR vs. CON IUGR

Comparison of gene methylation status showed that there was a greatly different methylation pattern between the CON_IUGR and CON_NBW groups. Most of these DMRs were observed on the upstream and downstream 2-kb genes. Many genes were enriched for hypomethylation in IUGR, such as transcription regulation, development process, and cell surface genes (Fig. 5). Some genes were significantly down-regulated in IUGR. Methylated genes tended to be enriched in transcription factor activity and folic acid binding. With respect to the biological processes, the genes were enriched in functions associated with response to hormone, cell adhesion, insulin receptor signaling pathway, endothelial cell proliferation, and fat cell differentiation (Fig. 5A). Further research on these genes may elucidate the functions of hypomethylation in the ileum of the IUGR piglets. Compared to the MET_NBW group, gene methylation status in the MET_IUGR group was decreased, and hypomethylated genes tended to be enriched in calcium iron binding in the molecular function category and extracellular space in the cellular component category (Fig. 5B). Gene methylation status comparison showed that there was a different methylation pattern between the MET_IUGR and CON_IUGR groups. The up-regulated methylated genes tended to be enriched in the growth hormone receptor signaling pathway via the JAK-STAT signaling pathway, ER-associated misfolded protein, transforming growth factor beta-activated receptor activity, and nitric oxide (NO) biosynthetic process. Significantly enriched down-regulated genes included insulin-like growth factor I binding, fatty acid beta-oxidation, calmodulin-dependent protein kinase activity, calcium ion-binding, and DNA-binding transcription factor activity (Fig. 5C).

## Discussion

Genetic improvement in sow husbandry over the course of 10 years has resulted in 1.8 extra piglets per litter and a reduction in birth weight by 180 g [2]. These results showed that maternal MET supplementation during gestation reduced the incidence of LBW piglets and increased the piglets’ weaning weight. Maternal diet enriched with MET was associated with increased fetal weight in late gestation [29], as well as higher BW at birth, weaning, and finishing stages [15, 30]. Although folic acid supplementation during pregnancy had no significant effect on the reproductive performance of sows [31], folic acid and vitamin B_12_ significantly reduced the incidence of early pregnancy failure in sows [32]. Interestingly, a study conducted in fathers found that there was a negative association between birth weight and betaine/methionine intake [33]. Consistently, higher maternal betaine status in women during gestation was associated with reduced birth weight [34]. However, protein restriction and methyl donor excess impaired postnatal growth and long-term weight gain in the offspring [35]. Therefore, appropriate MET levels might promote fetal development and improve postnatal growth performance. However, MET deficiency or excess and unbalanced nutritional environment may have adverse effects on the growth and development of the offspring. The MET complex components that have leading roles should be explored in the future.

Intestinal morphology, such as VH and CD, can reflect the intestinal development and function. In the present study, the IUGR piglets exhibited a decrease in VH and VCR and an increase in CD in the ileum, whereas MET supplementation and BW had an interactive effect on CD, which was consistent with previous study results [7]. Santos et al. [5] found that histomorphometrical parameters were not markedly affected during the pre-weaning period, and that the most detrimental effects on the small intestine histomorphometry of the duodenum epithelium were noticed at 70 days of age. Furthermore, the results showed that the birth weight had no significant effect on the activities of maltase and sucrase in the jejunum of newborn piglets, but the birth weight and MET supplementation had an interactive effect on the activity of lactase in the jejunum, which is consistent with the outcomes of a previous study [26, 36]. It was reported that differential intestinal disaccharidase activity between NBW and IUGR rabbit fetuses was observed, and both lactase and maltase activities were decreased in the IUGR fetuses that continued into the neonatal period [37]. Even the IUGR pigs demonstrated the lowest chymotrypsin and amylase activities at 70 and 150 days of age, respectively [5]. Finally, the addition of methyl donors to sow diets significantly increased the activities of lactase and sucrase in the jejunum of newborn piglets and improved the intestinal digestive function of newborn IUGR piglets. Our previous study also found that maternal MET supplementation significantly increased jejunum lactase activity and up-regulated the mRNA expression of jejunum Pept1 and lactase in the offspring [38]. Studies have also found that maternal folic acid supplementation during gestation increased the expression of *IGF-I* and sodium-glucose cotransporters in newborn lambs and improved the development of the offspring’s small intestine [39]. Additionally, betaine supplementation increased the trypsin activity of the jejunum in heat stressed broilers [40]. Therefore, the increases in lactase and sucrase activities contributed to improving the growth performance during lactation of offspring with maternal MET supplementation.

The present study results indicate that maternal MET supplementation during gestation reduces the total plasma amino acid and urea concentrations, suggesting that the utilization efficiency of amino acids in sows can be improved. A prior study reported that asparagine, cystathionine, and threonine in porcine milk were affected by maternal dietary choline intake [41]. Furthermore, choline supplementation did not alter methionine-homocysteine metabolism, but resulted in increased glycine and decreased threonine, histidine, valine, and total branch chained amino acid levels in children [42]. Betaine is involved in the synthesis of methionine from homocysteine in the liver. Betaine intake in male mice increased the methionine, *S*-adenosylmethionine, and *S*-adenosylhomocysteine levels and decreased the homocysteine and cystathionine levels [43]. Rumen-protected betaine supplementation increased the content of total free amino acids and flavor amino acids in the *longissimus dorsi* of lambs [44]. Folate plays a critical role in DNA replication and methylation and is involved in amino acid metabolism. Liu et al. [45] reported that folic acid supplementation during pregnancy influences maternal proline and γ-aminobutyric acid levels and leucine, isoleucine, serine, and proline levels in rat pups. In addition, metabolic changes in the offspring in terms of maternal folic acid supplementation is characterized by changes in the levels of tryptophan, glycine, and γ-aminobutyric acid [46]. In addition, plasma cysteine concentration was markedly increased by the activity of methionine synthase, which also increased by 14%–27% in the ileum of IUGR piglets in the present study. Methionine synthase is involved in re-methylation of homocysteine to methionine. Increased methionine synthase level may explain the lower homocysteine level reported in the IUGR groups, which is closely linked to IUGR [47]. Based on these results, amino acid alterations in mothers and their offspring are associated with maternal MET supplementation and may be involved in intestinal development, which warrants future research.

Differences in both gene expression and DNA methylation patterns in the intestine may indicate changes in intestinal development and function. Research has shown that IUGR can cause DNA methylation abnormalities [12], lower expression of genes involved in nutrient digestion and barrier function, and delayed development of intestinal villi and crypts [48], which lead to gastrointestinal dysfunction. Although there were no global-scale differences in DNA methylation between the IUGR and NBW piglets, divergently methylated regions were observed. Relative to their NBW littermates, the IUGR piglets had much more DMC hypomethylation in the ileum, suggesting that the global ileum epigenome dysfunction might be linked to the degree of IUGR impairment. The changes in methylation observed in the key regulators of intestinal development in the LBW piglets suggested long-term effects of BW on intestinal gene expression, development, and function [48]. Similarly, IUGR changes were noted in cytosine methylation at ∼1,400 loci in pancreatic islets of male rats at 7 weeks of age [49]. However, little research has been conducted on the effect of MET supplementation on methylation in the ileum and intestinal tract. Liu et al. [50] reported that IUGR impaired intestinal development, while maternal folic acid supplementation dramatically increased DNMT-1 and Bcl-2 expression and decreased apoptosis-related gene expression in the jejunum section of the IUGR piglets. Consistently, there were high hypomethylation levels in folic acid binding between the CON_IUGR and CON_ NBW groups, which implies that folic acid metabolism is one possible reason for lower methylation in the IUGR piglets. Furthermore, it was reported that early life (in utero) folate depletion affects epigenetic marking, while post-weaning folate supply does not significantly affect small intestine genomic DNA methylation [51]. However, dietary betaine hydrochloride addition to sow diets increased the VH and VH to CD ratio in the jejunum and ileum of suckling piglets [52]. Choline functions as a methyl group donor in a pathway that produces *S*-adenosylmethionine. Research has shown that gestational choline addition in mice modulates the expression of DNA (*Dnmt1* and *Dnmt3a*) and histone (G9a/Ehmt2/Kmt1c and Suv39h1/Kmt1a) methyltransferases [53]. After 10 weeks, colonic DNA in B_12_ rats demonstrated a 35% decrease in genomic methylation and a 105% increase in uracil incorporation in the colonic epithelium [54]. Children with lower concentrations of B_6_, B_9_, and B_12_ may have methylation deficiency [55]. Moreover, B_12_ addition in ileal epithelial cells sustained cellular methylation programs, leading to differential CpG methylation of genes associated with intestinal barrier function and cell proliferation [56]. Interestingly, B_12_ deficiency in combination with folate deficiency led to an increase in mRNA levels of *DNMT1* in all fetal tissues, whereas no effect was observed in combination with normal folate levels. Additionally, B_12_ over-supplementation combined with either state of folate level did not affect fetal DNMT1 expression [57].

There was a significant change in methylation in the IUGR group after MET supplementation. Increased hypermethylated genes were enriched in the hormone receptor pathway via JAK-STAT, nitric-oxide biosynthetic process, and dopamine receptor signaling pathway. It has been reported that NO exerts a wide range of protective and anti-inflammatory effects on the intestine [58]. Pre-treatment of mice with NO significantly alleviated small intestinal damage induced by indomethacin, as demonstrated by the down-regulation of pro-inflammatory cytokine and chemokine CXCL1/KC levels [59]. Intestinal NO metabolism and synthesis was altered in the small intestine of aging mice. L-Arginine is the biological precursor of NO and treatment with an arginase inhibitor prevents aging-associated intestinal barrier dysfunction and low-grade endotoxemia [60]. A similar result showed that D_2_ dopamine receptor binding-related protein levels were reduced in the IUGR piglets [61]. Fecal dopamine level in F344 rats was elevated in association with acute stress, which implies that dopamine level may be relevant in intestinal pathophysiology [62]. Furthermore, it was reported that maternal MET supplementation during gestation alleviated the adverse effects induced by bisphenol A. This includes the suppression of intestinal digestion and absorption function in the offspring, which might be associated with up-regulated mRNA expression of jejunum *DNMT1* and *DNMT3a*, as well their DNA methylation level [38]. Supplementation with resveratrol and curcumin in weaning piglets has been shown to increase growth performance and enhance intestinal function, which may be associated with changes in m^6^A methylation and gene expression [63]. Maternal hydroxytyrosol supplementation starting on d 35 of pregnancy and lasting until d 100 of pregnancy in sows increased DNA methylation in the fetuses [64].

## Conclusion

In conclusion, IUGR can lead to abnormal intestinal DNA methylation in a pig model. This alteration in DNA methylation can be alleviated by maternal MET supplementation by regulating the related gene expression and their function in various biological processes, such as NO biosynthetic process, hormone receptor pathway via JAK-STAT, and dopamine receptor signaling pathway. These findings may provide clues for the improvement of IUGR in the swine industry and in human patients.

## Data Availability

All data generated or analyzed during this study are included.

## Abbreviations

BHMT: Betaine homocysteine methyltransferase
BW: Body weight
CD: Crypt depth
CON: Control group
DFI: Daily feed intake
DMCs: Differentially methylated CpG
DMRs: Differentially methylated regions
DNMT: DNA methyltransferase
IUGR: Intrauterine growth restriction
MET: Methyl donors
MetS: Methionine synthase
NBW: Normal birth weight
NO: Nitric oxide
RRBS: Reduced representation bisulfite sequencing
VH: Villus height

## References

[1] Sharma D, Shastri S, Sharma P (2016). Intrauterine growth restriction: antenatal and postnatal aspects. Clin Med Insights Pediatr.

[2] Quesnel H, Brossard L, Valancogne A, Quiniou N (2008). Influence of some sow characteristics on within-litter variation of piglet birth weight. Animal.

[3] Yang YSH, Chou HC, Liu YR, Chen CM (2022). Uteroplacental insufficiency causes microbiota disruption and lung development impairment in growth-restricted newborn rats. Nutrients.

[4] Che L, Hu L, Zhou Q, Peng X, Liu Y, Luo Y (2020). Microbial insight into dietary protein source affects intestinal function of pigs with intrauterine growth retardation. Eur J Nutr.

[5] Santos TG, Fernandes SD, de Oliveira Araújo SB, Felicioni F, de Mérici Domingues e Paula T, Caldeira-Brant AL, et al. Intrauterine growth restriction and its impact on intestinal morphophysiology throughout postnatal development in pigs. Sci Rep. 2022;12:11810. 10.1038/s41598-022-14683-z. 10.1038/s41598-022-14683-z PMC9276813 35821501

[6] Stremming J, Chang EI, Knaub LA, Armstrong AL, Baker PR, Wesolowski SR (2022). Lower citrate synthase activity, mitochondrial complex expression, and fewer oxidative myofibers characterize skeletal muscle from growth-restricted fetal sheep. Am J Physiol Regul Integr Comp Physiol.

[7] Wang T, Huo YJ, Shi F, Xu RJ, Hutz RJ (2005). Effects of intrauterine growth retardation on development of the gastrointestinal tract in neonatal pigs. Biol Neonate.

[8] Tang X, Xiong K. Intrauterine growth retardation affects intestinal health of suckling piglets via altering intestinal antioxidant capacity, glucose uptake, tight junction, and immune responses. Oxidative Med Cell Longev. 2022:2644205. 10.1155/2022/2644205. 10.1155/2022/2644205 PMC8957421 35345830

[9] Zhang LL, Zhang H, Li Y, Wang T (2017). Effects of medium-chain triglycerides on intestinal morphology and energy metabolism of intrauterine growth retarded weanling piglets. Arch Anim Nutr.

[10] Zhang H, Chen Y, Li Y, Wang T. Protective effect of polydatin on jejunal mucosal integrity, redox status, inflammatory response, and mitochondrial function in intrauterine growth-retarded weanling piglets. Oxidative Med Cell Longev. 2020:7178123. 10.1155/2020/7178123. 10.1155/2020/7178123 PMC7576365 33101591

[11] Zhang W, Ma C, Xie P, Zhu Q, Wang X, Yin Y (2019). Gut microbiota of newborn piglets with intrauterine growth restriction have lower diversity and different taxonomic abundances. J Appl Microbiol.

[12] Hu Y, Hu L, Gong D, Lu H, Xuan Y, Wang R (2018). Genome-wide DNA methylation analysis in jejunum of Sus scrofa with intrauterine growth restriction. Mol Gen Genomics.

[13] Almeida MM, Dias-Rocha CP, Reis-Gomes CF, Wang H, Atella GC, Cordeiro A (2019). Maternal high-fat diet impairs leptin signaling and up-regulates type-1 cannabinoid receptor with sex-specific epigenetic changes in the hypothalamus of newborn rats. Psychoneuroendocrinology.

[14] Sulaiman SA, De Blasio MJ, Harland ML, Gatford KL, Owens JA (2017). Maternal methyl donor and cofactor supplementation in late pregnancy increases β-cell numbers at 16 days of life in growth-restricted twin lambs. Am J Physiol Endocrinol Metab.

[15] Jin C, Zhuo Y, Wang J, Zhao Y, XuanY LH (2018). Methyl donors dietary supplementation to gestating sows diet improves the growth rate of offspring and is associating with changes in expression and DNA methylation of insulin-like growth factor-1 gene. J Anim Physiol Anim Nutr.

[16] Pauwels S, Duca RC, Devlieger R, Freson K, Straetmans D, Van Herck E (2016). Maternal methyl-group donor intake and global DNA (Hydroxy)methylation before and during pregnancy. Nutrients.

[17] Sinclair KD, Allegrucci C, Singh R, Gardner DS, Sebastian S, Bispham J (2007). DNA methylation, insulin resistance, and blood pressure in offspring determined by maternal periconceptional B vitamin and methionine status. Proc Natl Acad Sci U S A.

[18] King JH, Kwan STC, Yan J, Jianget X, Fominal VG, Levine SP (2019). Maternal choline supplementation modulates placental markers of inflammation, angiogenesis, and apoptosis in a mouse model of placental insufficiency. Nutrients.

[19] Bossenmeyer-Pourié C, Blaise S, Pourié G, Tomasetto C, Audonnet S, Ortiou S (2010). Methyl donor deficiency affects fetal programming of gastric ghrelin cell organization and function in the rat. Am J Pathol.

[20] Hu M, Suter M, Bocock P, Shope C, Showalter L, Aagaard-Tillery K (2010). FGF21 is epigenetically regulated by a methyl donor rich diet and a transgenerational model of IUGR. Biol Reprod.

[21] Dasso JF, Hu M, Shope C, Showalter L, Lane R, Aagaard K (2011). Epigenetic regulation of insulin-like growth factor-1 by a methyl donor-rich diet in a transgenerational model of intrauterine growth restriction. Biol Reprod.

[22] Gonzalez-Rodriguez P, Cantu J, O'Neil D, Seferovic MD, Goodspeed DM, Suter MA (2016). Alterations in expression of imprinted genes from the H19/IGF2 loci in a multigenerational model of intrauterine growth restriction (IUGR). Am J Obstet Gynecol.

[23] NRC (2012). Nutrient requirements of swine.

[24] Xiong B, Pang Z, Zhao F, Luo Q. Chinese feed databases. The table of Chinese feed ingredient and its nutritional values (25th ed). China Feed. 2014;21:29–39.

[25] Che L, Zhou Q, Liu Y, Hu L, Peng X, Wu C (2019). Flaxseed oil supplementation improves intestinal function and immunity, associated with altered intestinal microbiome and fatty acid profile in pigs with intrauterine growth retardation. Food Funct.

[26] Che L, Hu L, Liu Y, Yan C, Peng X, Xu Q (2016). Dietary nucleotides supplementation improves the intestinal development and immune function of neonates with intra-uterine growth restriction in a pig model. PLoS One.

[27] Xi Y, Li W (2009). BSMAP: whole genome bisulfite sequence MAPping program. BMC Bioinformatics.

[28] Jühling F, Kretzmer H, Bernhart SH, Otto C, Stadler PF, Hoffmann S (2016). Metilene: fast and sensitive calling of differentially methylated regions from bisulfite sequencing data. Genome Res.

[29] Oster M, Nuchchanart W, Trakooljul N, Muráni E, Zeyner A, Wirthgen E (2016). Methylating micronutrient supplementation during pregnancy influences foetal hepatic gene expression and IGF signalling and increases foetal weight. Eur J Nutr.

[30] He Q, Zou T, Chen J, Jian L, He J, Xia Y (2020). Maternal methyl-donor micronutrient supplementation during pregnancy promotes skeletal muscle differentiation and maturity in newborn and weaning pigs. Front Nutr.

[31] Harper AF, Knight JW, Kokue E, Usry JL (2003). Plasma reduced folates, reproductive performance, and conceptus development in sows in response to supplementation with oxidized and reduced sources of folic acid. J Anim Sci.

[32] Van Wettere W, Smits RJ, Hughes PE (2012). Methyl donor supplementation of gestating sow diets improves pregnancy outcomes and litter size. Anim Prod Sci.

[33] Pauwels S, Truijen I, Ghosh M, Duca RC, Langie SAS, Bekaert B (2017). The effect of paternal methyl-group donor intake on offspring DNA methylation and birth weight. J Dev Orig Health Dis.

[34] Du YF, Wei Y, Yang J, Cheng ZY, Zuo XF, Wu TC (2019). Maternal betaine status, but not that of choline or methionine, is inversely associated with infant birth weight. Br J Nutr.

[35] Giudicelli F, Brabant AL, Grit I, Parnet P, Amarger V (2013). Excess of methyl donor in the perinatal period reduces postnatal leptin secretion in rat and interacts with the effect of protein content in diet. PLoS One.

[36] Niu Y, Zhao YW, He JT, Shen MM, Gan ZD, Zhang LL (2020). Dietary dihydroartemisinin supplementation improves growth, intestinal digestive function and nutrient transporters in weaned piglets with intrauterine growth retardation. Livest Sci.

[37] Buchmiller-Crair TL, Gregg JP, Rivera FA, Choi RS, Diamond JM, Fonkalsrud EW (2001). Delayed disaccharidase development in a rabbit model of intrauterine growth retardation. Pediatr Res.

[38] Liu H, Wang J, Mou D, Che L, Fang Z, Feng B (2017). Maternal methyl donor supplementation during gestation counteracts the bisphenol a-induced impairment of intestinal morphology, disaccharidase activity, and nutrient transporters gene expression in newborn and weaning pigs. Nutrients.

[39] Li Z, Wang B, Li H, Jian L, Luo H, Wang B (2020). Maternal folic acid supplementation differently affects the small intestinal phenotype and gene expression of newborn lambs from differing litter sizes. Animals.

[40] Liu W, Yuan Y, Sun C, Balasubramanian B, Zhao Z, An L (2019). Effects of dietary betaine on growth performance, digestive function, carcass traits, and meat quality in indigenous yellow-feathered broilers under long-term heat stress. Animals.

[41] Mudd AT, Alexander LS, Johnson SK, Getty CM, Malysheva OV, Caudill MA (2016). Perinatal dietary choline deficiency in sows influences concentrations of choline metabolites, fatty acids, and amino acids in milk throughout lactation. J Nutr.

[42] Alshaikh B, Schall JI, Maqbool A, Mascarenhas M, Bennett MJ, Stallings VA (2016). Choline supplementation alters some amino acid concentrations with no change in homocysteine in children with cystic fibrosis and pancreatic insufficiency. Nutr Res.

[43] Kim SK, Kim YC (2005). Effects of betaine supplementation on hepatic metabolism of sulfur-containing amino acids in mice. J Hepatol.

[44] Dong L, Zhong ZX, Cui HH, Wang NS, Luo Y, Yu LH (2020). Effects of rumen-protected betaine supplementation on meat quality and the composition of fatty and amino acids in growing lambs. Animal.

[45] Liu Z, Liu R, Chou J, Yu J, Liu X, Sun C (2018). Targeted metabolomics analysis reveals the association between maternal folic acid supplementation and fatty acids and amino acids profiles in rat pups. J Chromatogr B Analyt Technol Biomed Life Sci.

[46] Liu L, Liu Z, Li Y, Sun C (2021). Integration of metabolomics and proteomics to highlight altered neural development related pathways in the adult offspring after maternal folic acid supplement. Clin Nutr.

[47] Infante-Rivard C, Rivard GE, Gauthier R, Théorêt Y (2003). Unexpected relationship between plasma homocysteine and intrauterine growth restriction. Clin Chem.

[48] Ayuso M, Irwin R, Walsh C, Van Cruchten S, Van Ginneken C (2021). Low birth weight female piglets show altered intestinal development, gene expression, and epigenetic changes at key developmental loci. FASEB J.

[49] Thompson RF, Fazzari MJ, Niu H, Barzilai N, Simmons RA, Greally JM (2010). Experimental intrauterine growth restriction induces alterations in DNA methylation and gene expression in pancreatic islets of rats. J Biol Chem.

[50] Liu J, Chen D, Mao X, Yu B (2011). Effects of maternal folic acid supplementation on morphology and apoptosis-related gene expression in jejunum of newborn intrauterine growth retarded piglets. Arch Anim Nutr.

[51] McKay JA, Waltham KJ, Williams EA, Mathers JC (2011). Folate depletion during pregnancy and lactation reduces genomic DNA methylation in murine adult offspring. Genes Nutr.

[52] Azad MAK, Gao Q, Ma C, Wang K, Kong X (2022). Betaine hydrochloride addition in Bama mini-pig's diets during gestation and lactation enhances immunity and alters intestine microbiota of suckling piglets. J Sci Food Agric.

[53] Blusztajn JK, Mellott TJ (2012). Choline nutrition programs brain development via DNA and histone methylation. Cent Nerv Syst Agents Med Chem.

[54] Choi SW, Friso S, Ghandour H, Bagley PJ, Selhub J, Mason JB (2004). Vitamin B-12 deficiency induces anomalies of base substitution and methylation in the DNA of rat colonic epithelium. J Nutr.

[55] Belardo A, Gevi F, Zolla L (2019). The concomitant lower concentrations of vitamins B_6_, B_9_ and B_12_ may cause methylation deficiency in autistic children. J Nutr Biochem.

[56] Ge Y, Zadeh M, Mohamadzadeh M (2022). Vitamin B_12_ regulates the transcriptional, metabolic, and epigenetic programing in human ileal epithelial cells. Nutrients.

[57] Mahajan A, Sapehia D, Thakur S, Mohanraj PS, Bagga R, Kaur J (2019). Effect of imbalance in folate and vitamin B_12_ in maternal/parental diet on global methylation and regulatory miRNAs. Sci Rep.

[58] Wallace JL (2019). Nitric oxide in the gastrointestinal tract: opportunities for drug development. Br J Pharmacol.

[59] Wang X, Shi J, Xu Z, Wang D, Song Y, Han G (2023). Targeted delivery of nitric oxide triggered by α-glucosidase to ameliorate NSAIDs-induced enteropathy. Redox Biol.

[60] Brandt A, Baumann A, Hernández-Arriaga A, Jung F, Nier A, Staltner R (2022). Impairments of intestinal arginine and no metabolisms trigger aging-associated intestinal barrier dysfunction and 'inflammaging'. Redox Biol.

[61] Huang S, Liu C, Li N, Wu Z, Li T, Han D (2020). Membrane proteomic analysis reveals the intestinal development is deteriorated by intrauterine growth restriction in piglets. Funct Integr Genomics.

[62] Bauer EE, Reed CH, Lyte M, Clark PJ (2022). An evaluation of the rat intestinal monoamine biogeography days following exposure to acute stress. Front Physiol.

[63] Gan Z, Wei W, Wu J, Zhao Y, Zhang L, Wang T (2019). Resveratrol and curcumin improve intestinal mucosal integrity and decrease m^6^A RNA methylation in the intestine of weaning piglets. ACS Omega.

[64] Garcia-Contreras C, Vazquez-Gomez M, Barbero A, Pesantez JL, Zinellu A, Berlinguer F (2019). Polyphenols and IUGR pregnancies: effects of maternal hydroxytyrosol supplementation on placental gene expression and fetal antioxidant status, dna-methylation and phenotype. Int J Mol Sci.

